# Prevalence and correlates of central venous catheter use among haemodialysis patients in the Irish health system - a national study

**DOI:** 10.1186/s12882-018-0873-x

**Published:** 2018-04-02

**Authors:** Wael F. Hussein, Husham Mohammed, Leonard Browne, Liam Plant, Austin G. Stack

**Affiliations:** 1 Department of Nephrology, University Hospital Limerick, St Nessans Rd, Dooradoyle, Limerick, Ireland; 2 Graduate Entry Medical School, University of Limerick, Limerick, Ireland; 3 National Renal Office, HSE Clinical Programmes and Strategy Division, Dublin, Ireland; 4 Department of Renal Medicine, University College Cork, Cork, Ireland; 5 Health Research Institute, University of Limerick, Limerick, Ireland

**Keywords:** Haemodialysis, Access, Arteriovenous fistula, Central venous catheter, Tunnelled dialysis catheter

## Abstract

**Background:**

Central venous catheters (CVC) are associated with substantial morbidity and mortality among patients undergoing haemodialysis (HD), yet they are frequently used as the primary vascular access for many patients on HD. The goal of this study was to determine the prevalence and variation in CVC use across centres in the Irish health system.

**Methods:**

Data from the National Kidney Disease Clinical Patient Management System (KDCPMS) was used to determine CVC use and patterns across centres. Data on demographic characteristics, primary cause of end-stage kidney disease (ESKD), comorbid conditions, laboratory values and centre affiliation were extracted for adult HD patients (*n* = 1, 196) who were on dialysis for at least three months up to end of December 2016. Correlates of CVC use were explored using multivariable logistic regression.

**Results:**

Overall prevalence of CVC use was 54% and varied significantly across clinical sites from 43% to 73%, *P* < 0.001. In multivariate analysis, the likelihood of CVC use was lower with increasing dialysis vintage, OR 0.40 (0.26–0.60) for 4 years vs 1 year vintage, rising serum albumin, OR 0.73 (0.59–0.90) per 5 g/L), and with cystic disease as a cause of ESKD, OR 0.38 (95% CI 0.21–0.6). In contrast, catheter use was greater for women than men, OR 1.77 (1.34–2.34) and for 2 out of 10 regional dialysis centres, OR 1.98 (1.02–3.84) and OR 2.86 (1.67–4.90) respectively compared to referent group).

**Conclusions:**

Catheters are the predominant type of vascular access in patients undergoing HD in the Irish health system. Substantial centre variation exists which is not explained by patient-level characteristics.

**Electronic supplementary material:**

The online version of this article (10.1186/s12882-018-0873-x) contains supplementary material, which is available to authorized users.

## Background

Patients who develop kidney failure and require haemodialysis (HD) experience substantial morbidity and mortality [1, 2]. It is well established that the type of permanent vascular access, central venous catheter (CVC), arteriovenous fistula (AVF) or graft (AVG), is a major determinant of subsequent morbidity and overall patient survival. For patients who are treated with HD, the risks of major cardiovascular events, fatal and non-fatal infections and overall mortality are far greater with catheters than with AV fistula [3–6]. Although the AV fistula is cited as the preferred access for most patients, a majority of patients are treated with tunnelled dialysis catheters [7–9]. In response, recommendations from professional societies and governmental organisations, along with clinical guidelines from expert groups, have advocated strongly for changes in clinical practice that would favour greater utilization of the AV fistula over catheter use in order to improve patient outcomes [10–12].

Observational studies, both national and international, have demonstrated substantial variation in vascular access utilisation among HD patients [9, 13–16]. Evidence suggests that both patient-level characteristics and facility-related factors contribute to the observed variation in vascular access provision [14–19]. The extent to which each set of factors contribute to the overall extent of variation in utilisation is unclear. On one hand, there is good evidence to invoke patient-level characteristics such as age, sex, race, presence of medical conditions, and patient preference as important determinants of access type [9]. On the other hand, emerging evidence suggests that variation in the rates of vascular access across HD centres persist even after correction of patient-related factors and implicate factors external to the patients such as process of care and lack of comprehensive multidisciplinary team programs [16]. A detailed understanding of the utilisation of vascular access within health systems in fundamental to strategic planning and organisation of access services in order to improve patients outcomes.

National data on the epidemiology of vascular access (VA) is lacking within the Irish health system, which may hamper strategic decision-making on the organisation and delivery of optimal vascular access care. A recent cross-sectional study by McCann et al. found that the prevalence of tunnelled dialysis catheters across Irish dialysis centres was 52%, ranging from 35 to 80% across participating centres [20]. The survey identified deficiencies in the organisation and delivery of vascular access provision including lack of dedicated theatre time for vascular surgery as contributing factors. Recently, Ireland, as part of its national renal strategy, has implemented a national patient electronic system, *the* “*Kidney Disease Clinical Patient Management System (KDCPMS)”*, in order to track the care and outcomes of patients with moderate to advanced CKD within the health system. This system has provided a unique opportunity to gain greater insight into the provision of HD access care in the Irish health system and facilitate studies to explore variation across centres and to support quality improvement initiatives.

The goal of this study was to describe the prevalence of vascular access among prevalent HD patients in the Irish health system and examine for variation in access use across clinical sites. Moreover, we sought to determine whether any observed variation could be explained by differences in measureable demographic, clinical and dialysis-related factors.

## Methods

### Clinical setting

Renal replacement therapies in the Republic of Ireland are provided through 11 primary kidney centres, organised into six hospital groups. Under supervision of these primary centres, haemodialysis (HD) for adult patients is organised and supervised across 20 dialysis units. Every patient on HD has a “centre of primary medical supervision” at which their care is managed, including arrangements for provision of vascular access placement.

KDCPMS is a kidney-specific national information system that tracks patients from late-stage CKD across the transition to end-stage kidney disease (ESKD) across all dialysis centres in Ireland. The system interfaces directly with local hospital information systems to capture real-time data on demographics and laboratory results for individual patients. Data such as primary cause of ESKD, comorbid conditions, medications, and ancillary notes on clinical care delivery are manually entered by users at site of care. Each renal centre has a local KDCPMS supervisor, whose responsibilities include user management, data reporting and quality control.

Over the last several years, renal centres in Ireland were incrementally added on KDCPMS. In 2017, all units in the Republic of Ireland were included in the system. At the end of December 2016, the observation time-point for this paper, 17 HD facilities from 10 centres of primary medical supervision, had fully enrolled in KDCPMS.

### Study design and data sources

**Fig. 1:**
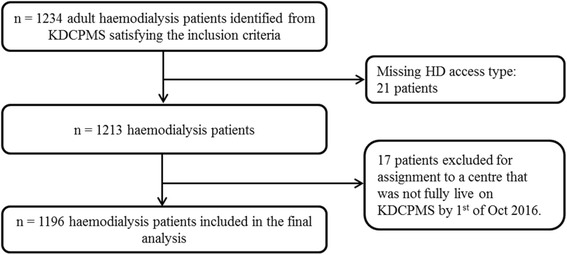
Flow chart of study cohort. KDCPMS: Kidney Disease Clinical Patient Management System

We conducted a national cross-sectional study of vascular access among adult prevalent HD patients who were alive and active on HD on 31/12/2016 at KDCPMS-participating centres for at least 3 months - Fig. 1. We included patients who received a minimum of 6 dialysis treatment sessions per month from October to December 2016. Patients who had their primary medical supervision at a site that was not active on KDCPMS were excluded. Patients with no recorded access type at any session in November and December 2016 were also excluded.

Data were captured from KDCPMS on demographic characteristics, comorbid conditions, primary cause of ESKD, vascular access type, location of medical supervision, and monthly laboratory values for all HD patients during the study period. The type of vascular access recorded for each patient between November 1st and December 31st 2016 was retrieved, and vascular access assignment was based on the last recorded access used for HD in this period. Primary cause of kidney disease and comorbid diagnoses were collapsed into categories and classified as per the US Renal Data System. A patient was considered to have hypertension or diabetes if these conditions were listed among the comorbid conditions or if diabetes or hypertension was among the causes of kidney disease. Laboratory variables measured between 1/9/2016 and 31/12/2016 were retrieved and the time-weighted median value for each test type was determined and included in the final dataset. Ten clinical sites that were active on KDCPMS in 2016 were included in this study. For the purposes of this study, patients who were treated with an AVG (*n* = 14) were grouped with those using an AVF. Patient assignment to a specific dialysis centre was based on last “location of primary medical supervision” during 2016. Ethical approval was not sought as this study was part of a quality improvement initiative and satisfied the ethical and information governance for analysis of secondary health data for improvement in population health [21].

### Statistical methods

Baseline characteristics were described for the whole population, by type of access, and by HD centre, as counts and proportion for categorical variables, and as means and standard deviations for continuous variables. The number of patients in each centre was suppressed to maintain centre anonymity. The Chi-square and ANOVA tests were used to test for differences between groups as appropriate. Missing data were imputed using predictive mean matching with 50 iterations, generating 5 complete datasets. Multivariable logistic regression was used to explore factors associated with CVC use versus AVF. The explanatory variables were classified as demographic, primary cause of ESKD, comorbid medical conditions, laboratory indicators of health, and dialysis centre. Model building progressed in a manual fashion based on univariate associations, clinical reasoning and previous published literature. A final model was constructed to explore the relative contribution of all explanatory factors with the presence of catheters. The associations of explanatory factors with catheter presence were represented by adjusted odds-ratios (AOR) and 95% CI. For each model, the C-statistic was calculated to assess the model performance. Several sensitivity analyses were performed to assess the robustness of our results. First, we repeated the analysis using the first recorded result for each laboratory value (within the period from 1st of November to 31st of December 2016) instead of the weighted medians described above. Second, we re-analysed the data using only patients with complete data on all measured variables (complete case analysis). The final analytic dataset was constructed and analysed using R version 3.4.

## Results

From a total of 1234 patients satisfying the inclusion criteria, 38 patients were excluded, and the remaining 1196 patients were included in the analysis (Fig. 1).

### Descriptive statistics

The baseline characteristics of the study population by type of vascular access are shown in Table 1. Overall, 54% of the patients received dialysis through CVC. The mean age was 65 years, 31% of the patients were 75 years of age or older, and 37% of patients were female. The prevalence of diabetes and hypertension were 31% and 56% respectively. Patients with CVC had a higher proportion of older patients and females. Moreover, a higher proportion of patients were dialysing with a CVC rather than AVF in the first year of dialysis compared to later vintage periods.

**Table 1:** Patient characteristics by type of vascular access

Variable Mean (SD) or Frequency %	Overall	Arteriovenous Fistula	Central Venous Catheter	*p*-value
(*n* = 1196)				
Vascular Access Type		45.7%	54.3%	
**Demographic Characteristic (%)**				
Age (year)	64.8 (15.1)	63.2 (15.2)	66.1 (14.9)	0.0009
Age Group				0.0138
< 65	42.7	47.0	39.1	
65–74	26.5	25.8	27.1	
75+	30.8	27.2	33.7	
Female	36.9	30.0	42.7	< 0.0001
**Lifestyle Factor (%)**				
Body mass index (kg/m^2^)	27.3 (6.5)	27.6 (6.1)	27.1 (6.8)	0.1960
BMI Group				0.0315
< 20	4.8	3.2	6.2	
20–25	34.8	33.0	36.5	
26–30	33.4	36.6	30.6	
> 30	26.9	27.2	26.7	
**Comorbid conditions (%)**				
Hypertension	56.4	58.9	54.2	0.1212
Diabetes mellitus	31.4	29.1	33.4	0.1191
Atherosclerotic heart disease	18.6	18.1	19.1	0.7105
Congestive heart disease	15.7	15.4	16	0.8130
Other cardiac disease	15.7	15.4	16	0.8130
Cerebrovascular disease	7.3	5.9	8.5	0.1033
Peripheral vascular disease	5.7	5.9	5.5	0.9202
**Primary Kidney Disease (%)**				< 0.0001
Glomerulonephritis	19.2	20.3	18.3	
Diabetes	18.6	18.5	18.8	
Hypertension	8.6	10.2	7.2	
Cystic kidney disease	6.4	9.5	3.7	
Other urologic	9.4	9.5	9.4	
Other cause	9.5	9.5	9.6	
Unknown/missing	28.2	22.5	33.0	
**Laboratory values (mean (SD))**				
Albumin (g/L)	36.8 (4.4)	37.5 (3.9)	36.3 (4.7)	< 0.0001
Calcium (mmol/L)	2.3 (0.2)	2.3 (0.2)	2.2 (0.2)	0.0025
Phosphorous (mmol/L)	1.6 (0.4)	1.6 (0.4)	1.5 (0.4)	0.3880
Pre-dialysis creatinine (μmol/L)	731.1 (243.7)	776.8 (236.2)	692.8 (243.5)	< 0.0001
**Dialysis Vintage (%)**				< 0.0001
< 1 year	15.6	9.7	20.7	
1–4 years	42.6	43.5	41.9	
> 4 years	41.7	46.8	37.4	

Percentage of missing data: Body mass index (and BMI group): 11.2%, Albumin 21.7%, Calcium 21.6%, Phosphorous 21.6%, Pre-dialysis creatinine 7.5%, Dialysis Vintage: 0.6%

The median number of patients per centre was 103 (IQR: 91–147). The distribution of baseline characteristics across dialysis centres is shown in Additional file 1: Table S1. CVC use varied significantly across participating dialysis centres, ranging from 43% to 73%, *P* < 0.0001. Significant variation also existed across clinical sites in relation to age, recorded comorbid medical conditions, primary kidney disease, laboratory values and vintage groups.

### Association of Patient-level Characteristics with CVC use

The relationship between demographic and lifestyle factors, and comorbid conditions with CVC use was explored in a series of univariable and multivariable models of increasing complexity. In the unadjusted models, increasing age and female sex were significantly associated with CVC use (Table 2). Patients with cystic kidney disease as primary cause of ESKD (compared to glomerulonephritis, referent) experienced significantly lower odds of CVC use while patients with unknown or missing case of ESKD had significantly higher odds ratio. Serum albumin and pre-dialysis creatinine concentrations, both nutritional indicators of health, were associated with significantly lower odds of CVC. The findings from the univariate analysis were confirmed in the multivariable analysis. Women were significantly more likely to have a CVC as their principal access than men (OR 1.77, 95% CI 1.34–2.34), and this association persisted following adjustment for age, comorbid indicators, and lifestyle factors. Similarly, patients with ESKD from cystic diseases of the kidney experienced lower odds of CVC compared to those with a primary glomerulonephritis (OR 0.38, 95% CI 0.21–0.68). There was a strong inverse association of dialysis vintage with CVC use such that patients on HD for 4 years or more had a 60% lower odds of having a CVC (OR 0.40, 9R% CI 0.26–0.60).

**Table 2:** Factors associated with Central Venous Catheter Use (vs Fistula) in the Irish Health System (Odds Ratios and 95% Confidence Intervals) – Univariate Analysis

Variable	OR (95% Confidence Intervals)
**Demographic factors**	
Female (vs male)	1.74 (1.37–2.21)****
Age group (years)	
< 65 (Referent)	1.00
65–74	1.26 (0.95–1.67)
75 +	1.49 (1.13–1.95)**
**Lifestyle Factor**	
Body mass index (BMI) group (kg/m^2^)	
< 20	1.65 (0.88–3.12)
20–25 (Referent)	1.00
25–30	0.78 (0.58–1.04)
> 30	0.83 (0.61–1.12)
**Comorbid conditions**	
Atherosclerotic heart disease (yes vs no)	1.07 (0.8–1.43)
Diabetes (yes vs no)	1.23 (0.96–1.57)
Hypertension (yes vs no)	0.83 (0.66–1.04)
**Primary cause of kidney disease**	
Glomerulonephritis (Referent)	1.00
Cystic kidney disease	0.43 (0.25–0.75) **
Diabetes	1.13 (0.78–1.63)
Hypertension	0.78 (0.49–1.25)
Other cause of ESKD	1.11 (0.71–1.75)
Other urologic disease	1.09 (0.7–1.72)
Unknown/missing	1.62 (1.15–2.28) **
**Laboratory values**	
Albumin (per 5 g/L)	0.71 (0.61–0.83)****
Pre-dialysis Creatinine (per 50 μmol/L)	0.93 (0.91–0.95)****
**Vintage (years)**	
< 1 (Referent)	1.00
1–4	0.45 (0.31–0.65)****
4+	0.37 (0.26–0.53)****

Asterisks denote significant *p* values: * < 0.05, ** < 0.01, *** < 0.001, **** < 0.0001

### Association of Dialysis Centre with CVC use

**Fig. 2:**
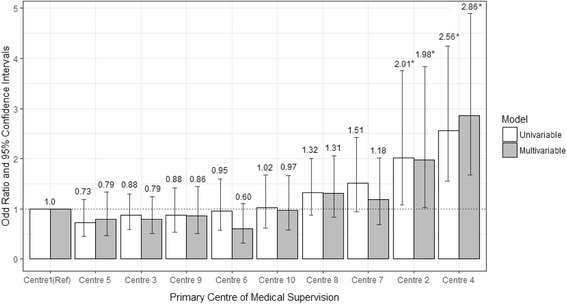
Odd ratios and 95% confidence intervals for dialysis by central venous catheters (versus arteriovenous fistulas) among centres in the Irish health system. Multivariable model adjusted for demographic factors: sex (female versus male), age group (< 65 (Ref), 65–74 and > 75 years); lifestyle factors: body mass index group (< 20, 20–25 (Ref), 25–30 and > 30 kg/m^2^); vintage (< 1 (Ref), 1–4 and > 4 years); comorbid conditions (yes versus no for each): atherosclerotic heart disease, diabetes, hypertension; primary cause of kidney disease: glomerulonephritis (Ref), cystic kidney disease, diabetes, hypertension, other cause, other urologic, unknown/missing; and laboratory values: albumin (per 5 g/L) and pre-dialysis creatinine (per 50 μmol/L) . * denotes statistically significant results

The univariable models identified a strong association of dialysis centre (centre 2 and 4) with CVC use as shown in Table 3. With sequential adjustment for patient-level characteristics including dialysis vintage, Centres 2 and 4 experienced higher odds ratios of CVC use (OR 2.01, 95% CI: 1.08–3.75, and 2.56, 95% CI: 1.55–4.25 respectively) compared to the referent Centre 1. Surprisingly, the magnitude of the association was virtually unchanged following adjustment. Our analysis demonstrated that the dialysis centre had a significant contribution to the performance of all models, with a Wald test *p* value of < 0.001. However, our final model yielded a C-statistic of 0.69-moderate performance, suggesting that the final set of variables did not adequately explain variation in use. The association of dialysis centres with CVC use in the univariable model and the fully adjusted model is demonstrated in Fig. 2.

**Table 3:** Unadjusted and multivariable adjusted Odd Ratios and 95% confidence intervals for Central Venous Catheter use (vs Fistula)

Variable	Model 1	Model 2	Model 3	Model 4	Model 5
**Dialysis Centre**					
Centre 1 (Referent)	1.00	1.00	1.00	1.00	1.00
Centre 2	2.01 (1.08–3.75) *	1.86 (0.99–3.50)	1.91 (1.00–3.65)*	1.98 (1.02–3.81)*	1.98 (1.02–3.84)*
Centre 3	0.88 (0.59–1.30)	0.85 (0.57–1.26)	0.77 (0.51–1.16)	0.83 (0.54–1.26)	0.79 (0.51–1.24)
Centre 4	2.56 (1.55–4.25) ***	2.59 (1.55–4.33)***	2.59 (1.53–4.37)***	2.61 (1.54–4.43)***	2.86 (1.67–4.90)***
Centre 5	0.73 (0.45–1.18)	0.69 (0.42–1.14)	0.71 (0.43–1.18)	0.72 (0.43–1.20)	0.79 (0.47–1.33)
Centre 6	0.95 (0.57–1.60)	0.96 (0.57–1.63)	0.87 (0.51–1.50)	0.91 (0.52–1.58)	0.60 (0.32–1.11)
Centre 7	1.51 (0.94–2.43)	1.41 (0.87–2.29)	1.36 (0.83–2.24)	1.47 (0.88–2.45)	1.18 (0.69–2.01)
Centre 8	1.32 (0.87–2.00)	1.36 (0.89–2.07)	1.23 (0.79–1.91)	1.18 (0.75–1.85)	1.31 (0.83–2.06)
Centre 9	0.88 (0.54–1.42)	0.83 (0.51–1.36)	0.74 (0.45–1.23)	0.81 (0.48–1.35)	0.86 (0.51–1.45)
Centre 10	1.02 (0.62–1.68)	0.98 (0.59–1.62)	0.87 (0.52–1.47)	0.90 (0.53–1.52)	0.97 (0.57–1.66)
**Demographic Factor**					
Female (vs male)		1.68 (1.31–2.15)****	1.79 (1.39–2.31)****	1.87 (1.44–2.43)****	1.77 (1.34–2.34)****
Age group (years)					
< 65 (Ref)		1.00	1.00	1.00	1.00
65–74		1.30 (0.97–1.74)	1.20 (0.89–1.62)	1.19 (0.87–1.61)	1.05 0.76–1.45)
≥ 75		1.46 (1.10–1.94)**	1.41 (1.05–1.89)*	1.35 (1.00–1.83)	1.15 (0.83–1.60)
**Lifestyle factor**					
Body mass index (BMI) group (kg/m^2^)					
< 20		1.74 (0.91–3.36)	1.81 (0.93–3.53)	1.86 (0.95–3.62)	1.78 (0.91–3.50)
20–25 (Ref)		1.00	1.00	1.00	1.00
25–30		0.81 (0.60–1.09)	0.77 (0.57–1.04)	0.75 (0.55–1.02)	0.77 (0.56–1.05)
> 30		0.82 (0.60–1.13)	0.73 (0.52–1.02)	0.73 (0.52–1.03)	0.76 (0.54–1.08)
Vintage (years)					
< 1 (Ref)			1.00	1.00	1.00
1–4			0.45 (0.31–0.65)****	0.46 (0.31–0.67)****	0.48 0.33–0.71)***
> 4			0.36 (0.24–0.52)****	0.39 (0.26–0.57)****	0.40 (0.26–0.60)****
**Comorbid conditions**					
Atherosclerotic heart disease (yes vs no)			1.13 (0.81–1.56)	1.11 (0.80–1.55)	1.09 (0.78–1.52)
Diabetes (yes vs no)			1.40 (1.06–1.86)*	1.50 (1.02–2.23)*	1.38 (0.93–2.06)
Hypertension (yes vs no)			0.78 (0.60–1.01)	0.85 (0.65–1.11)	0.86 (0.65–1.13)
**Primary cause of kidney disease**					
Glomerulonephritis (Referent group)				1.00	1.00
Cystic kidney disease				0.37 (0.21–0.67)***	0.38 (0.21–0.68)**
Diabetes				0.84 (0.50–1.41)	0.85 (0.50–1.43)
Hypertension				0.77 (0.46–1.30)	0.83 (0.49–1.41)
Other cause				1.05 (0.65–1.70)	1.03 (0.63–1.69)
Other urologic				1.15 (0.71–1.85)	1.13 (0.70–1.85)
Unknown/missing				1.29 (0.89–1.87)	1.30 (0.89–1.90)
**Laboratory values**					
Albumin (per 5 g/L)					0.73 (0.59–0.90)**
Pre-dialysis Creatinine (per 50 μmol/L)					0.99 (0.96–1.03)
**Model performance**					
C-Statistic	0.59	0.64	0.67	0.68	0.69
P value of Centre variable	0.0002	0.0002	0.0001	0.0003	0.0001
ANOVA p value for model with and without Centre variable	–	0.0001	< 0.0001	< 0.0001	< 0.0001

Model 1: dialysis centre only

Model 2: dialysis centre, demographic and lifestyle characteristics (age, sex, and body mass index)

Model 3: dialysis centre, demographic and lifestyle characteristics, dialysis vintage, and comorbid conditions

Model 4: dialysis centre, demographic and lifestyle characteristics (age group, sex, and body mass index), dialysis vintage, and comorbid conditions, and primary cause of kidney disease

Model 5: dialysis centre, demographic and lifestyle characteristics (age group, sex, and body mass index), dialysis vintage, and comorbid conditions, and primary cause of kidney disease, and laboratory indicators (serum albumin, and creatinine measured pre-dialysis)

Asterisks denote significant *p* values: * < 0.05, ** < 0.01, *** < 0.001, **** < 0.0001

## Discussion

In this nationally representative study, we describe for the first time the prevalence and correlates of CVC use in a contemporary cohort of HD patients in the Irish health system. Central venous catheters were the predominant type of vascular access, with a prevalence of 54%. We found that women were significantly more likely than men to have a CVC in-situ adjusting for case-mix, and that CVC use became less frequent with increasing dialysis vintage. Within the health system, we observed substantial variation in CVC use across dialysis centres that ranged from 43 to 73%. Quite strikingly these differences across centres did not diminish when adjustment was made for baseline differences in demographic characteristics and comorbid indicators of illness such that in the final model, 2 out of 10 dialysis centres continued to experience a 2–3 fold higher use of CVC than AVF compared to the reference centre. These findings would suggest that additional factors, such as patient preference, and the structure and organisation of care delivery prior to and after dialysis initiation, contribute to differences in vascular access provision and utilisation within the Irish health system.

Despite introduction of evidence-based guidelines and strategic initiatives by many countries to improve the provision of AVF and reduce dependency on CVC, this study in the Irish health system highlights substantial dependence on CVC by a majority of HD patients. There is good evidence to suggest that better pre-dialysis care, more effective organisation of care by multidisciplinary teams, and incentivisation schemes lead to greater creation of AVF over CVCs [22–25]. The fistula first initiative in the US is an excellent example where national policy underpinned by a strong regulatory framework has led a significant increase in AVF use and overall reduction in CVC [26–28]. In Ireland, McCann and colleagues reported a CVC prevalence of 52% in 2011 from results of a national survey of vascular access [20]. A direct comparison with our results 5 years later, highlight similar prevalence, suggesting that little has changed. Internationally, CVC use varies significantly among countries. Among centres participating in the International Dialysis Outcomes and Practice Patterns Study (IDOPPS) between August 2010 and August 2013, CVC prevalence ranged from 6% in Japan, up to 45% in Canada. In Europe, Germany had the lowest CVC prevalence (15%), while the highest prevalence was noted in Belgium (38%) [9]. Our prevalence estimate of 54% in Ireland is one of the highest in Europe, and suggests that further attention be given to identifying risk factors and to establishing a framework for corrective action.

The current study draws further attention to centre-to-centre variation in the clinical practice of vascular access provision. It is widely acknowledged that some of this variation in prevalence may be attributed to variation in the “case-mix” of patients with sicker multi-morbid patients more likely to receive a CVC than an AVF. Several patient-related characteristics, including advanced age, female sex, low serum albumin and comorbid diseases such as diabetes, are associated with high CVC use [14–16, 29]. Indeed, our multivariable models, confirmed the relationship of many of these factors with CVC use. We considered that differences in the distribution of these patient-level characteristics across clinical sites might explain some of the observed variation. However, quite surprisingly, adjusting for these factors in multivariable models did not attenuate the effect of dialysis centre on CVC use. The persistence of variation across clinical sites would suggest that factors other than comorbidity burden operate to influence the placement of permanent vascular access. The structure, organisation, and delivery of HD vascular access care is a complex process and requires multidisciplinary team engagement with defined care pathways and embedded quality assurance. The survey by McCann et al. identified a number of deficits in the health system including lack of availability of elective surgical beds and operating room slots, insufficient vascular access coordinators and access protocols which are likely to impact the timely and effective creation of AVFs [20]. Physician perspectives, as well as availability and experience of a vascular surgeon may also play a role. Internationally, geographical variability and local facility factors have been recognized as contributing factors [14–16].

Our study had some limitations that deserve mention. Unmeasured and residual confounding are inherent shortcomings given the retrospective nature of the study and may have contributed to our findings. We acknowledge that other explanatory factors known to affect fistula placement and maturation such as vessel size were not available in the dataset, and thus not accounted for. We also submit that while the cross-sectional nature of the study allowed us to determine prevalence, it limits the evaluation of the temporal sequence of events and thus we cannot determine cause-effect relationships. These shortcomings were however counterbalanced by several strengths. First, our study was national in scope and thus generalizable to all HD patients within the Irish health system. Second, the KDCPMS is now the primary clinical data system for recording patient care across all dialysis centres in Ireland. Consequently, it allowed us to capture data on key patient-related variables including demographic characteristics, measures of comorbidity, dialysis vintage and principal vascular access in a standardised fashion. Our primary exposure variable, vascular access type, was determined in real-time from dialysis treatments which ensured precision. Our observed rates for primary causes of kidney disease and prevalence of reported comorbid conditions in our cohort were similar to those reported in other European countries [30, 31]. Finally, in sensitivity analysis where we included patients with complete data on all variables, the findings were materially unchanged (see results in Additional file 2).

## Conclusions

CVCs are the predominant type of vascular access for Irish patients undergoing chronic HD. These high rates of CVC use have not changed over the past 5 years. Substantial variation in CVC use exists across dialysis centres, which is not explained by measurable patient-related factors. Findings from this study would suggest that additional factors, such as patient preference, limited availability of multidisciplinary vascular access teams, and lack of timely access to vascular surgical services contribute to the high rates of CVC use. Concerted efforts at a local and national level, informed by national policy, and international best practice are required to optimize fistula creation in appropriate patients. We hope that the findings from this national study will serve as a catalyst to engage all stakeholders and drive quality improvement in vascular access.

## Additional files

Additional file 1: **Table S1.** Patient Characteristics Across Dialysis Centres within the Irish Health System. (DOC 116 kb)

Additional file 2: Sensitivity analyses. (DOC 205 kb)

## Abbreviations

AOR: Adjusted odds ratio
AVF: Arteriovenous fistula
AVG: Arteriovenous graft
CVC: Central venous catheter
ESKD: End-stage kidney disease
HD: Haemodialysis
IQR: Interquartile range
KDCPMS: Kidney Disease Clinical Patient Management System
VA: Vascular access
