# Supplementary material for: Prevalence and correlates of central venous catheter use among haemodialysis patients in the Irish health system - a national study
Source: BMC Nephrol. 2018 Apr 2;19:76. doi: 10.1186/s12882-018-0873-x (PMC5880000; doi:10.1186/s12882-018-0873-x)
Supplement: Supplementary file 1 — Table S1. Patient Characteristics Across Dialysis Centres within the Irish Health System. (DOC 116 kb) [file 12882_2018_873_MOESM1_ESM.doc]

**Additional file 1: Table S1**: Patient Characteristics Across Dialysis Centres within the Irish Health System

| **Variable** | **Cohort**  n = 1196 | **Centre 1** | **Centre 2** | **Centre 3** | **Centre 4** | **Centre 5** | **Centre 6** | **Centre 7** | **Centre 8** | **Centre 9** | **Centre 10** | **p-value** |
| --- | --- | --- | --- | --- | --- | --- | --- | --- | --- | --- | --- | --- |
| **Vascular Access (%)** |  |  |  |  |  |  |  |  |  |  |  | 0.0001 |
| Arteriovenous fistula | 45.7 | 48.8 | 32.1 | 52.1 | 27.1 | 56.6 | 50.0 | 38.7 | 41.9 | 52.1 | 48.3 |  |
| Central venous catheter | 54.3 | 51.2 | 67.9 | 47.9 | 72.9 | 43.4 | 50.0 | 61.3 | 58.1 | 47.9 | 51.7 |  |
| **Demographic Factors** |  |  |  |  |  |  |  |  |  |  |  |  |
| Age in years (mean, (SD)) | 64.8 (15.1) | 62.0 (15.5) | 68.5 (13) | 64.8 (13.8) | 67.2 (15.5) | 67.5 (15.7) | 63 (13.1) | 66.3 (15) | 63 (15.7) | 65.9 (16.1) | 65.2 (15.1) | 0.0104 |
| **Age Group (%)** |  |  |  |  |  |  |  |  |  |  |  | 0.0280 |
| <65 | 42.7 | 50.7 | 30.4 | 43.8 | 36.4 | 37.4 | 53.8 | 37.7 | 44.4 | 38.5 | 40.4 |  |
| 65-74 | 26.5 | 23 | 32.1 | 30.4 | 25.2 | 28.3 | 27.5 | 23.6 | 28.7 | 20.8 | 27 |  |
| 75+ | 30.8 | 26.3 | 37.5 | 25.8 | 38.3 | 34.3 | 18.8 | 38.7 | 26.9 | 40.6 | 32.6 |  |
| **Gender** |  |  |  |  |  |  |  |  |  |  |  |  |
| Female (%) | 36.9 | 39.2 | 42.9 | 33.0 | 36.4 | 32.3 | 36.2 | 44.3 | 32.5 | 39.6 | 38.2 | 0.5600 |
| **Lifestyle Factor** |  |  |  |  |  |  |  |  |  |  |  |  |
| Body mass index (kg/m^2^) | 27.3 (6.5) | 27.9 (6.9) | 27.6 (5.8) | 26.0 (5.8) | 28.6 (6.3) | 26.6 (5.9) | 27.2 (9.2) | 26.7 (5.7) | 27.2 (5.9) | 27.3 (6) | 27.8 (6.7) | 0.1330 |
| BMI Group |  |  |  |  |  |  |  |  |  |  |  | 0.4596 |
| <20 | 4.8 | 4.0 | 6.0 | 9.2 | 1.0 | 6.0 | 5.0 | 4.1 | 1.9 | 4.2 | 8.3 |  |
| 20-25 | 34.8 | 32.8 | 30.0 | 39.0 | 30.6 | 38.1 | 38.8 | 35.1 | 39.0 | 32.3 | 28.6 |  |
| 26-30 | 33.4 | 34.3 | 36.0 | 29.1 | 37.8 | 36.9 | 32.5 | 35.1 | 32.5 | 34.4 | 28.6 |  |
| >30 | 26.9 | 28.9 | 28.0 | 22.7 | 30.6 | 19.0 | 23.8 | 25.7 | 26.6 | 29.2 | 34.5 |  |
| **Comorbid conditions (%)** |  |  |  |  |  |  |  |  |  |  |  |  |
| Hypertension | 56.4 | 62.2 | 82.1 | 38.1 | 71.0 | 81.8 | 65.0 | 46.2 | 33.8 | 66.7 | 53.9 | <0.0001 |
| Diabetes mellitus | 31.4 | 32.5 | 30.4 | 28.4 | 38.3 | 41.4 | 37.5 | 21.7 | 18.8 | 42.7 | 33.7 | 0.0002 |
| Atherosclerotic heart disease | 18.6 | 17.7 | 21.4 | 10.3 | 27.1 | 23.2 | 21.2 | 16.0 | 11.9 | 26.0 | 27.0 | 0.0007 |
| Congestive heart disease | 15.7 | 17.2 | 14.3 | 8.8 | 18.7 | 16.2 | 23.8 | 12.3 | 11.2 | 21.9 | 22.5 | 0.0113 |
| Other cardiac disease | 15.7 | 17.2 | 14.3 | 8.8 | 18.7 | 16.2 | 23.8 | 12.3 | 11.2 | 21.9 | 22.5 | 0.0113 |
| Cerebrovascular disease | 7.3 | 5.3 | 8.9 | 4.6 | 8.4 | 11.1 | 10.0 | 8.5 | 6.2 | 9.4 | 6.7 | 0.5472 |
| Peripheral Vascular Disease | 5.7 | 8.6 | 3.6 | 2.6 | 2.8 | 4.0 | 10.0 | 3.8 | 3.8 | 12.5 | 6.7 | 0.0072 |
| **Primary Kidney Disease** |  |  |  |  |  |  |  |  |  |  |  | <0.0001 |
| Glomerulonephritis | 19.2 | 16.7 | 16.1 | 28.4 | 23.4 | 17.2 | 17.5 | 17 | 17.5 | 11.5 | 20.2 |  |
| Diabetes | 18.6 | 17.2 | 14.3 | 21.1 | 25.2 | 18.2 | 22.5 | 14.2 | 10.6 | 32.3 | 13.5 |  |
| Hypertension | 8.6 | 8.1 | 8.9 | 4.6 | 9.3 | 16.2 | 3.8 | 18.9 | 4.4 | 9.4 | 7.9 |  |
| Cystic kidney disease | 6.4 | 6.7 | 10.7 | 7.2 | 2.8 | 5.1 | 10.0 | 5.7 | 3.8 | 9.4 | 5.6 |  |
| Other urologic | 9.4 | 15.3 | 3.6 | 9.3 | 7.5 | 10.1 | 8.8 | 13.2 | 6.9 | 4.2 | 7.9 |  |
| Other cause | 9.5 | 6.2 | 3.6 | 13.4 | 5.6 | 12.1 | 12.5 | 9.4 | 3.1 | 9.4 | 23.6 |  |
| Unknown/ missing | 28.2 | 29.7 | 42.9 | 16.0 | 26.2 | 21.2 | 25.0 | 21.7 | 53.8 | 24.0 | 21.3 |  |
| **Laboratory values** |  |  |  |  |  |  |  |  |  |  |  |  |
| Albumin (g/L) | 36.8 (4.4) | 37.2 (3.6) | 36.8 (3.7) | NA | 38.2 (3.6) | 38.6 (3.4) | 30.9 (4.0) | 33.7 (4.8) | 39.1 (2.8) | 38.0 (3.3) | 38.0 (4.0) | <0.0001 |
| Calcium (mmol/L) | 2.3 (0.2) | 2.3 (0.2) | 2.2 (0.1) | NA | 2.3 (0.1) | 2.2 (0.1) | 2.2 (0.2) | 2.2 (0.2) | 2.3 (0.2) | 2.3 (0.1) | 2.3 (0.1) | <0.0001 |
| Phosphorous (mmol/L) | 1.6 (0.4) | 1.5 (0.4) | 1.6 (0.4) | NA | 1.5 (0.3) | 1.5 (0.4) | 1.4 (0.4) | 1.7 (0.4) | 1.8 (0.4) | 1.5 (0.4) | 1.6 (0.4) | <0.0001 |
| Pre-dialysis Creatinine (µmol/L) | 731.1 (243.7) | 742.1 (239.7) | 683.8 (264.5) | 839.8 (250.6) | 676.4 (243.4) | 672.2 (214.8) | 695.2 (193.5) | 699.2 (255.4) | 802.8 (255.5) | 665.7 (240.3) | 742.4 (194.4) | <0.0001 |
| **Vintage (%)** |  |  |  |  |  |  |  |  |  |  |  | 0.0022 |
| <1 years | 15.6 | 12.0 | 16.4 | 15.5 | 16.0 | 10.1 | 21.2 | 16.0 | 17.7 | 18.1 | 18.0 |  |
| 1-4 years | 42.6 | 36.4 | 36.4 | 44.6 | 36.8 | 36.4 | 33.8 | 48.1 | 55.1 | 45.7 | 47.2 |  |
| 4+ years | 41.7 | 51.7 | 47.3 | 39.9 | 47.2 | 53.5 | 45.0 | 35.8 | 27.2 | 36.2 | 34.8 |  |

Footnote: NA: None available
