# Supplementary material for: Prevalence and correlates of central venous catheter use among haemodialysis patients in the Irish health system - a national study
Source: BMC Nephrol. 2018 Apr 2;19:76. doi: 10.1186/s12882-018-0873-x (PMC5880000; doi:10.1186/s12882-018-0873-x)
Supplement: Supplementary file 2 — Sensitivity analyses. (DOC 205 kb) [file 12882_2018_873_MOESM2_ESM.doc]

Sensitivity Analysis for

*Prevalence and Correlates of Central Venous Catheter Use Among Haemodialysis Patients in the Irish Health System - A National Study*

Wael F Hussein, Husham Mohammed, Leonard Browne, Liam Plant, Austin G Stack

Univariable analysis:

- Logistic regression results for variables with missing values. Odd ratio and 95% confidence intervals for complete case analysis and on imputed dataset.

| **Variable** | **Imputed dataset**  **OR (95% Confidence Intervals)** | **Complete case analysis**  **OR (95% Confidence Intervals)** |
| --- | --- | --- |
| **Lifestyle Factor** |  |  |
| Body mass index (BMI) group (kg/m^2^) |  |  |
| < 20 | 1.65 (0.88 - 3.12) | 1.76 (0.96 - 3.37) |
| 20 – 25 (Referent) | 1.00 | 1.00 |
| 25 – 30 | 0.78 (0.58 - 1.04) | 0.76 (0.56 - 1.01) |
| > 30 | 0.83 (0.61 - 1.12) | 0.89 (0.65 - 1.21) |
| **Laboratory values** |  |  |
| Albumin (per 5 g/L) | 0.71 (0.61 - 0.83)**** | 0.71 (0.61 - 0.83)**** |
| Pre-dialysis Creatinine (per 50 µmol/L) | 0.93 (0.91 - 0.95)**** | 0.93 (0.91 - 0.95)**** |
| **Vintage (years)** |  |  |
| < 1 (Referent) | 1.00 | 1.00 |
| 1 – 4 | 0.45 (0.31 - 0.65)**** | 0.45 (0.31 - 0.64)**** |
| > 4 | 0.37 (0.26 - 0.53)**** | 0.37 (0.26 - 0.53)**** |

Footnote: Asterisks denote significant p values: * < 0.05, ** <0.01, *** <0.001, **** <0.0001

First Lab instead of weighted median – univariate analysis

| **Laboratory values** | With weighted median results | With first result |
| --- | --- | --- |
| Albumin (per 5 g/L) | 0.71 (0.61 - 0.83)**** | 0.73 ( 0.60 - 0.88)** |
| Pre-dialysis Creatinine (per 50 µmol/L) | 0.93 (0.91 - 0.95)**** | 0.94 (0.92 - 0.96)**** |

Multivariable analysis: Comparing results of the five constructed models using three datasets:

1. Imputed data, weighted means for lab values
2. Imputed data, first lab result for each test for each patient
3. Complete case analysis, with number of patients included shown for cases with complete data for the variables used in the model.

Model 1: Odd ratio and 95% confidence intervals of receiving dialysis by central venous catheter by dialysis centre.

| **Variable** | **Using weighted median for laboratory values**  N = 1196 | **Using first result for laboratory values**  N = 1196 | **Complete case analysis**  **N =** 1196 |
| --- | --- | --- | --- |
| **Dialysis Centre** |  |  |  |
| Centre 1 (Referent) | 1.00 | 1.00 | 1.00 |
| Centre 2 | 2.01 (1.08 - 3.75) * | 2.01 (1.08 - 3.75)* | 2.01 (1.09 - 3.82)* |
| Centre 3 | 0.88 (0.59 - 1.30) | 0.88 (0.59 - 1.30) | 0.88 (0.59 - 1.3) |
| Centre 4 | 2.56 (1.55 - 4.25) *** | 2.56 (1.55 - 4.25)*** | 2.56 (1.56 - 4.3)*** |
| Centre 5 | 0.73 (0.45 - 1.18) | 0.73 (0.45 - 1.18) | 0.73 (0.45 - 1.18) |
| Centre 6 | 0.95 (0.57 - 1.60) | 0.95 (0.57 - 1.60) | 0.95 (0.57 - 1.6) |
| Centre 7 | 1.51 (0.94 - 2.43) | 1.51 (0.94 - 2.43) | 1.51 (0.94 - 2.44) |
| Centre 8 | 1.32 (0.87 – 2.00) | 1.32 (0.87 - 2.00) | 1.32 (0.87 - 2.01) |
| Centre 9 | 0.88 (0.54 - 1.42) | 0.88 (0.54 - 1.42) | 0.88 (0.54 - 1.42) |
| Centre 10 | 1.02 (0.62 - 1.68) | 1.02 (0.62 - 1.68) | 1.02 (0.62 - 1.68) |

Model 2: Odd ratio and 95% confidence intervals of receiving dialysis by central venous catheter by dialysis centre, adjusted for sex, age group and body mass index.

| **Variable** | **Using weighted median for laboratory values**  N = 1196 | **Using first result for laboratory values**  N = 1196 | **Complete case analysis**  N **=** 1062 |
| --- | --- | --- | --- |
| **Dialysis Centre** |  |  |  |
| Centre 1 (Referent) | 1.00 | 1.00 | 1.00 |
| Centre 2 | 1.86 (0.99 - 3.50) | 1.85 (0.98 - 3.49) | 2.16 (1.12 - 4.34)* |
| Centre 3 | 0.85 (0.57 - 1.26) | 0.85 (0.57 - 1.27) | 0.76 (0.49 - 1.18) |
| Centre 4 | 2.59 (1.55 - 4.33)*** | 2.58 (1.55 - 4.32)*** | 2.39 (1.43 - 4.06)** |
| Centre 5 | 0.69 (0.42 - 1.14) | 0.69 (0.42 - 1.14) | 0.65 (0.38 - 1.1) |
| Centre 6 | 0.96 (0.57 - 1.63) | 0.97 (0.57 - 1.63) | 1 (0.59 - 1.7) |
| Centre 7 | 1.41 (0.87 - 2.29) | 1.41 (0.87 - 2.29) | 1.11 (0.64 - 1.91) |
| Centre 8 | 1.36 (0.89 - 2.07) | 1.36 (0.89 - 2.07) | 1.43 (0.93 - 2.2) |
| Centre 9 | 0.83 (0.51 - 1.36) | 0.83 (0.51 - 1.36) | 0.87 (0.53 - 1.43) |
| Centre 10 | 0.98 (0.59 - 1.62) | 0.97 (0.59 - 1.62) | 1.09 (0.65 - 1.83) |
| **Demographic Factor** |  |  |  |
| Female (vs male) | 1.68 (1.31 - 2.15)**** | 1.68 (1.31 - 2.16)**** | 1.55 (1.19 - 2.02)** |
| Age group (years) |  |  |  |
| < 65 (Ref) | 1.00 | 1.00 | 1.00 |
| 65 – 74 | 1.30 (0.97 - 1.74) | 1.30 (0.97 - 1.74) | 1.31 (0.96 - 1.78) |
| >75 | 1.46 (1.10 - 1.94)** | 1.46 (1.10 - 1.94)** | 1.37 (1.02 - 1.86)* |
| **Lifestyle factor** |  |  |  |
| Body mass index (BMI) group (kg/m^2^) |  |  |  |
| < 20 | 1.74 (0.91 - 3.36) | 1.64 (0.86 - 3.14) | 1.91 (1.02 - 3.71)* |
| 20 – 25 (Ref) | 1.00 | 1.00 | 1.00 |
| 25 – 30 | 0.81 (0.60 - 1.09) | 0.79 (0.59 - 1.07) | 0.78 (0.57 - 1.05) |
| > 30 | 0.82 (0.60 - 1.13) | 0.82 (0.60 - 1.12) | 0.87 (0.63 - 1.2) |

Model 3: Odd ratio and 95% confidence intervals of receiving dialysis by central venous catheter by dialysis centre, adjusted for sex, age group, body mass index, vintage and comorbid conditions.

| **Variable** | **Using weighted median for laboratory values**  N = 1196 | **Using first result for laboratory values**  N = 1196 | **Complete case analysis**  **N =** 1055 |
| --- | --- | --- | --- |
| **Dialysis Centre** |  |  |  |
| Centre 1 (Referent) | 1.00 | 1.00 | 1.00 |
| Centre 2 | 1.91 (1.00 - 3.65)* | 1.90 (1.00 - 3.62) | 2.13 (1.08 - 4.34)* |
| Centre 3 | 0.77 (0.51 - 1.16) | 0.77 (0.51 - 1.17) | 0.69 (0.44 - 1.09) |
| Centre 4 | 2.59 (1.53 - 4.37)*** | 2.58 (1.53 - 4.35)*** | 2.36 (1.39 - 4.05)** |
| Centre 5 | 0.71 (0.43 - 1.18) | 0.71 (0.43 - 1.17) | 0.65 (0.38 - 1.12) |
| Centre 6 | 0.87 (0.51 - 1.50) | 0.87 (0.51 - 1.49) | 0.90 (0.52 - 1.55) |
| Centre 7 | 1.36 (0.83 - 2.24) | 1.35 (0.82 - 2.22) | 1.17 (0.67 - 2.05) |
| Centre 8 | 1.23 (0.79 - 1.91) | 1.23 (0.79 - 1.91) | 1.29 (0.82 - 2.02) |
| Centre 9 | 0.74 (0.45 - 1.23) | 0.73 (0.44 - 1.22) | 0.74 (0.44 - 1.23) |
| Centre 10 | 0.87 (0.52 - 1.47) | 0.87 (0.52 - 1.47) | 0.99 (0.58 - 1.69) |
| **Demographic Factor** |  |  |  |
| Female (vs male) | 1.79 (1.39 - 2.31)**** | 1.78 (1.38 - 2.30)**** | 1.68 (1.28 - 2.20)*** |
| Age group (years) |  |  |  |
| < 65 (Ref) | 1.00 | 1.00 | 1.00 |
| 65 – 74 | 1.20 (0.89 - 1.62) | 1.20 (0.89 - 1.62) | 1.22 (0.89 - 1.67) |
| >75 | 1.41 (1.05 - 1.89)* | 1.42 (1.06 - 1.89)* | 1.32 (0.97 - 1.8) |
| **Lifestyle factor** |  |  |  |
| Body mass index (BMI) group (kg/m^2^) |  |  |  |
| < 20 | 1.81 (0.93 - 3.53) | 1.69 (0.88 - 3.25) | 1.97 (1.03 - 3.88)* |
| 20 – 25 (Ref) | 1.00 | 1.00 | 1.00 |
| 25 – 30 | 0.77 (0.57 - 1.04) | 0.76 (0.56 - 1.03) | 0.74 (0.54 - 1.01) |
| > 30 | 0.73 (0.52 - 1.02) | 0.73 (0.53 - 1.02) | 0.77 (0.55 - 1.08) |
| Vintage (years) |  |  |  |
| < 1 (Ref) | 1.00 | 1.00 | 1.00 |
| 1 – 4 | 0.45 (0.31 - 0.65)**** | 0.44 (0.30 - 0.64)**** | 0.46 (0.3 - 0.69)*** |
| > 4 | 0.36 (0.24 - 0.52)**** | 0.36 (0.24 - 0.52)**** | 0.38 (0.25 - 0.57)**** |
| **Comorbid conditions** |  |  |  |
| Atherosclerotic heart disease  (yes vs no) | 1.13 (0.81 - 1.56) | 1.13 (0.81 - 1.56) | 1.26 (0.89 - 1.79) |
| Diabetes  (yes vs no) | 1.40 (1.06 - 1.86)* | 1.40 (1.05 - 1.85)* | 1.42 (1.06 - 1.91)* |
| Hypertension  (yes vs no) | 0.78 (0.60 - 1.01) | 0.78 (0.60 - 1.01) | 0.76 (0.58 – 1.00) |

Model 4: Odd ratio and 95% confidence intervals of receiving dialysis by central venous catheter by dialysis centre, adjusted for sex, age group, body mass index, vintage, comorbid conditions and primary cause of kidney disease.

| **Variable** | **Using weighted median for laboratory values**  N = 1196 | **Using first result for laboratory values**  N = 1196 | **Complete case analysis**  N = 1055 |
| --- | --- | --- | --- |
| **Dialysis Centre** |  |  |  |
| Centre 1 (Referent) | 1.00 | 1.00 | 1.00 |
| Centre 2 | 1.98 (1.02 - 3.81)* | 1.97 (1.02 - 3.79)* | 2.22 (1.12 - 4.58)* |
| Centre 3 | 0.83 (0.54 - 1.26) | 0.83 (0.54 - 1.26) | 0.74 (0.46 - 1.17) |
| Centre 4 | 2.61 (1.54 - 4.43)*** | 2.60 (1.54 - 4.41)*** | 2.4 (1.41 - 4.14)** |
| Centre 5 | 0.72 (0.43 - 1.20) | 0.72 (0.43 - 1.19) | 0.67 (0.38 - 1.15) |
| Centre 6 | 0.91 (0.52 - 1.58) | 0.91 (0.52 - 1.58) | 0.95 (0.54 - 1.65) |
| Centre 7 | 1.47 (0.88 - 2.45) | 1.46 (0.88 - 2.43) | 1.26 (0.71 - 2.24) |
| Centre 8 | 1.18 (0.75 - 1.85) | 1.18 (0.75 - 1.85) | 1.25 (0.79 - 1.97) |
| Centre 9 | 0.81 (0.48 - 1.35) | 0.80 (0.48 - 1.34) | 0.81 (0.48 - 1.36) |
| Centre 10 | 0.90 (0.53 - 1.52) | 0.90 (0.53 - 1.52) | 1.00 (0.58 - 1.73) |
| **Demographic Factor** |  |  |  |
| Female (vs male) | 1.87 (1.44 - 2.43)**** | 1.86 (1.43 - 2.42)**** | 1.74 (1.32 - 2.3)**** |
| Age group (years) |  |  |  |
| < 65 (Ref) | 1.00 | 1.00 | 1.00 |
| 65 – 74 | 1.19 (0.87 - 1.61) | 1.19 (0.88 - 1.62) | 1.19 (0.86 - 1.65) |
| >75 | 1.35 (1.00 - 1.83) | 1.36 (1.00 - 1.84)* | 1.25 (0.91 - 1.73) |
| **Lifestyle factor** |  |  |  |
| Body mass index (BMI) group (kg/m^2^) |  |  |  |
| < 20 | 1.86 (0.95 - 3.62) | 1.74 (0.91 - 3.34) | 1.95 (1.01 - 3.89) |
| 20 – 25 (Ref) | 1.00 | 1.00 | 1.00 |
| 25 – 30 | 0.75 (0.55 - 1.02) | 0.74 (0.55 - 1.01) | 0.72 (0.53 - 0.99)* |
| > 30 | 0.73 (0.52 - 1.03) | 0.74 (0.53 - 1.03) | 0.78 (0.55 - 1.1) |
| Vintage (years) |  |  |  |
| < 1 (Ref) | 1.00 | 1.00 | 1.00 |
| 1 – 4 | 0.46 (0.31 - 0.67)**** | 0.45 (0.31 - 0.67)**** | 0.47 (0.31 - 0.72)*** |
| > 4 | 0.39 (0.26 - 0.57)**** | 0.39 (0.26 - 0.57)**** | 0.42 (0.27 - 0.63)**** |
| **Comorbid conditions** |  |  |  |
| Atherosclerotic heart disease  (yes vs no) | 1.11 (0.80 - 1.55) | 1.11 (0.80 - 1.55) | 1.23 (0.87 - 1.75) |
| Diabetes  (yes vs no) | 1.50 (1.02 - 2.23)* | 1.50 (1.01 - 2.22)* | 1.55 (1.03 - 2.37)* |
| Hypertension  (yes vs no) | 0.85 (0.65 - 1.11) | 0.84 (0.64 - 1.11) | 0.81 (0.6 - 1.08) |
| **Primary cause of kidney disease** |  |  |  |
| Glomerulonephritis  (Referent group) | 1.00 | 1.00 | 1.00 |
| Cystic kidney disease | 0.37 (0.21 - 0.67)*** | 0.37 (0.21 - 0.67)*** | 0.36 (0.19 - 0.67)** |
| Diabetes | 0.84 (0.50 - 1.41) | 0.84 (0.50 - 1.41) | 0.84 (0.48 - 1.45) |
| Hypertension | 0.77 (0.46 - 1.30) | 0.78 (0.46 - 1.31) | 0.83 (0.47 - 1.45) |
| Other cause | 1.05 (0.65 - 1.70) | 1.05 (0.65 - 1.70) | 1.12 (0.66 - 1.89) |
| Other urologic | 1.15 (0.71 - 1.85) | 1.14 (0.71 - 1.84) | 1.13 (0.67 - 1.89) |
| Unknown/missing | 1.29 (0.89 - 1.87) | 1.28 (0.88 - 1.86) | 1.29 (0.87 - 1.92) |

Model 5: Odd ratio and 95% confidence intervals of receiving dialysis by central venous catheter by dialysis centre, adjusted for sex, age group, body mass index, vintage, comorbid conditions, primary cause of kidney disease and laboratory values for serum albumin and predialysis creatinine.

| **Variable** | **Using weighted median for laboratory values**  N = 1196 | **Using first result for laboratory values**  N = 1196 | **Complete case analysis**  N = 847 |
| --- | --- | --- | --- |
| **Dialysis Centre** |  |  |  |
| Centre 1 (Referent) | 1.00 | 1.00 | 1.00 |
| Centre 2 | 1.98 ( 1.02 - 3.84)* | 1.96 ( 1.01 - 3.79)* | 2.42 (1.19 - 5.14)* |
| Centre 3 | 0.79 ( 0.51 - 1.24) | 0.80 ( 0.52 - 1.25) | - |
| Centre 4 | 2.86 ( 1.67 - 4.90)*** | 2.92 ( 1.70 - 5.01)*** | 2.59 (1.48 - 4.6)*** |
| Centre 5 | 0.79 ( 0.47 - 1.33) | 0.79 ( 0.47 - 1.34) | 0.74 (0.42 - 1.3) |
| Centre 6 | 0.60 ( 0.32 - 1.11) | 0.59 ( 0.32 - 1.11) | 0.61 (0.32 - 1.15) |
| Centre 7 | 1.18 ( 0.69 - 2.01) | 1.15 ( 0.67 - 1.98) | 0.97 (0.53 - 1.79) |
| Centre 8 | 1.31 ( 0.83 - 2.06) | 1.30 ( 0.83 - 2.06) | 1.09 (0.61 - 1.93) |
| Centre 9 | 0.86 ( 0.51 - 1.45) | 0.84 ( 0.50 - 1.42) | 0.83 (0.48 - 1.43) |
| Centre 10 | 0.97 ( 0.57 - 1.66) | 0.98 ( 0.57 - 1.68) | 1.01 (0.58 - 1.78) |
| **Demographic Factor** |  |  |  |
| Female (vs male) | 1.77 ( 1.34 - 2.34)**** | 1.81 ( 1.37 - 2.38)**** | 1.91 (1.37 - 2.66)*** |
| Age group (years) |  |  |  |
| < 65 (Ref) | 1.00 | 1.00 | 1.00 |
| 65 – 74 | - 1. 0.76 - 1.45) | 1.05 ( 0.77 - 1.45) | 1.19 (0.81 - 1.76) |
| >75 | 1.15 ( 0.83 - 1.60) | 1.15 ( 0.83 - 1.60) | 1.25 (0.85 - 1.85) |
| **Lifestyle factor** |  |  |  |
| Body mass index (BMI) group (kg/m^2^) |  |  |  |
| < 20 | 1.78 ( 0.91 - 3.50) | 1.58 ( 0.82 - 3.04) | 1.7 (0.78 - 3.9) |
| 20 – 25 (Ref) | 1.00 | 1.00 | 1.00 |
| 25 – 30 | 0.77 ( 0.56 - 1.05) | 0.76 ( 0.55 - 1.04) | 0.72 (0.5 - 1.03) |
| > 30 | 0.76 ( 0.54 - 1.08) | 0.75 ( 0.53 - 1.04) | 0.85 (0.57 - 1.25) |
| Vintage (years) |  |  |  |
| < 1 (Ref) | 1.00 | 1.00 | 1.00 |
| 1 – 4 | - 1. 0.33 - 0.71)*** | 0.49 ( 0.33 - 0.73)*** | 0.46 (0.28 - 0.76)** |
| > 4 | 0.40 ( 0.26 - 0.60)**** | 0.42 ( 0.28 - 0.63)**** | 0.39 (0.23 - 0.66)*** |
| **Comorbid conditions** |  |  |  |
| Atherosclerotic heart disease  (yes vs no) | 1.09 ( 0.78 - 1.52) | 1.08 ( 0.77 - 1.51) | 1.29 (0.87 - 1.93) |
| Diabetes  (yes vs no) | 1.38 ( 0.93 - 2.06) | 1.38 ( 0.92 - 2.06) | 1.64 (1.03 - 2.64)* |
| Hypertension  (yes vs no) | 0.86 ( 0.65 - 1.13) | 0.85 ( 0.65 - 1.12) | 0.76 (0.54 - 1.06) |
| **Primary cause of kidney disease** |  |  |  |
| Glomerulonephritis  (Referent group) | 1.00 | 1.00 | 1.00 |
| Cystic kidney disease | 0.38 ( 0.21 - 0.68)** | 0.38 ( 0.21 - 0.68)** | 0.4 (0.19 - 0.81)* |
| Diabetes | 0.85 ( 0.50 - 1.43) | 0.85 ( 0.50 - 1.44) | 0.8 (0.42 - 1.51) |
| Hypertension | 0.83 ( 0.49 - 1.41) | 0.84 ( 0.49 - 1.42) | 0.91 (0.49 - 1.69) |
| Other cause | 1.03 ( 0.63 - 1.69) | 1.02 ( 0.62 - 1.66) | 1.58 (0.85 - 2.96) |
| Other urologic | 1.13 ( 0.70 - 1.85) | 1.16 ( 0.71 - 1.88) | 1.17 (0.64 - 2.14) |
| Unknown/missing | 1.30 ( 0.89 - 1.90) | 1.31 ( 0.89 - 1.92) | 1.53 (0.96 - 2.43) |
| **Laboratory values** |  |  |  |
| Albumin (per 5 g/L) | 0.73 ( 0.59 - 0.90)** | 0.71 ( 0.56 - 0.90)** | 0.72 (0.57 - 0.9)** |
| Pre-dialysis Creatinine  (per 50 µmol/L) | 0.99 ( 0.96 - 1.03) | 0.99 ( 0.96 - 1.02) | 1.00 (0.96 - 1.04) |
